# Dynamics of epizootic hemorrhagic disease virus infection within the vector, *Culicoides sonorensis* (Diptera: Ceratopogonidae)

**DOI:** 10.1371/journal.pone.0188865

**Published:** 2017-11-27

**Authors:** Mary K. Mills, Mark G. Ruder, Dana Nayduch, Kristin Michel, Barbara S. Drolet

**Affiliations:** 1 Division of Biology, Kansas State University, Manhattan, Kansas, United States of America; 2 Southeastern Cooperative Wildlife Disease Study, University of Georgia, Athens, Georgia, United States of America; 3 United States Department of Agriculture, Agricultural Research Service, Arthropod-Borne Animal Diseases Research Unit, Manhattan, Kansas, United States of America; Onderstepoort Veterinary Institute, SOUTH AFRICA

## Abstract

*Culicoides sonorensis* biting midges are confirmed vectors of epizootic hemorrhagic disease virus (EHDV), which causes mortality in white-tailed deer and ruminant populations. Currently, of the seven EHDV serotypes, only 1, 2, and 6 are detected in the USA, and very few studies have focused on the infection time course of these serotypes within the midge. The objective of this current research was to characterize EHDV-2 infection within the midge by measuring infection prevalence, virus dissemination, and viral load over the course of infection. Midges were fed a blood meal containing 10^6.9^ PFU/ml EHDV-2, collected every 12 h from 0–2 days post feeding (dpf) and daily from 3–10 dpf, and cohorts of 20 *C*. *sonorensis* were processed using techniques that assessed EHDV infection and dissemination. Cytopathic effect assays and quantitative (q)PCR were used to determine infection prevalence, revealing a 50% infection rate by 10 dpf using both methods. Using immunohistochemistry, EHDV-2 infection was detectable at 5 dpf, and shown to disseminate from the midgut to other tissues, including fat body, eyes, and salivary glands by 5 dpf. Stain intensity increased from 5–8 dpf, indicating replication of EHDV-2 in secondary infection sites after dissemination. This finding is also supported by trends in viral load over time as determined by plaque assays and qPCR. An increase in titer between 4–5 dpf correlated with viral replication in the midgut as seen with staining at day 5, while the subsequent gradual increase in viral load from 8–10 dpf suggested viral replication in midges with disseminated infection. Overall, the data presented herein suggest that EHDV-2 disseminates via the hemolymph to secondary infection sites throughout the midge and demonstrate a high potential for transmission at five days at 25°C after an infective blood-meal.

## Introduction

*Culicoides* midges are hematophagous dipterans classified in the family Ceratopogonidae. Most *Culicoides* females take blood meals for egg production, making these insects nuisance biters and capable of vectoring a variety of pathogens [1], including arboviruses [2,3]. *Culicoides sonorensis* Wirth and Jones is a confirmed vector of the orbivirus (family Reoviridae) epizootic hemorrhagic disease (EHD) virus (EHDV) in the USA [4,5]. EHDV can cause disease in susceptible ruminant species, resulting in economic losses due to increased mortality within infected herds, production loss, and trade restrictions [6,7]. Historically, the geographic range of orbiviruses and their known *Culicoides* vectors was limited to between 35°S and 40°N; however, environmental change have resulted in an apparent increase in the vectorial capacity of populations in previously unfavourable areas [8–10]. In the USA, this range expansion has possibly played a role in more severe EHD outbreaks in susceptible herds from previously non-endemic areas, including a 2012 EHD outbreak in Michigan which resulted in the deaths of over 14,000 white-tailed deer [6,11,12]. In contrast, EHDV infection of white-tailed deer in the EHDV-endemic southern USA often results in mild infections with minimal incidence of mortality [13].

Despite this increase in sporadic EHD outbreaks, relatively little is known of the EHDV infection dynamics in *C*. *sonorensis*. Based on studies of *C*. *sonorensis* and bluetongue virus (BTV), orbivirus infection in *Culicoides* is limited by multiple barriers in its vector that have to be overcome for transmission to occur [14]. While BTV serotype 1 (BTV-1) was detected at 1 day post feeding (dpf) in all midges that had taken up a virus-containing blood meal, only a proportion of these midges developed persistent infection in the midgut, and only a small proportion of those midges with persistent midgut infections had BTV in their saliva. Thus, BTV encounters a midgut infection barrier (MIB) that limits virus entry and/or replication within the midgut epithelium and a midgut escape barrier (MEB), which the virus must overcome to disseminate through the hemolymph to secondary tissues [14–16]. In addition, Fu et al. [14] also observed a secondary dissemination barrier associated with the fat body. BTV was shown to penetrate the midge MEB as soon as 3 dpf [14,17], disseminate throughout the hemolymph, and infect the salivary glands as early as 5–7 dpf [14,18]. While a salivary gland barrier has been observed in multiple mosquito species [19–21], orbiviruses do not appear to encounter such a barrier in *C*. *sonorensis* midges [14,18,22].

Thus far, data on EHDV infection dynamics are sparse. A single study of EHDV has monitored the effects of temperature for two of the three EHDV serotypes within the USA (EHDV-1, -2, and -6) [23–26]. Ruder et al. [26] fed *C*. *sonorensis* midges a blood meal spiked with approximately 10^7.0^ TCID_50_/ml of EHDV-1, -2 and -7. EHDV-1 and -7 infected females had a high infection prevalence of approximately 90% throughout the experiment. In contrast, EHDV-2 infected midges had infection rates ranging from 25–30% at 1–2 dpf and 75–80% at 8–12 dpf. Titers ≥ 10^2.7^ TCID_50_/midge, the viral quantity threshold indicative of transmissible infection [14, 27, 28], were first observed at 6 dpf in EHDV-2 infected females at 25°C, and from 2–4 dpf at 30°C [26]. Overall, this study suggested that the extrinsic incubation period (EIP) required for these viruses to be transmitted after being ingested by the insect vector was highly temperature dependent [26]. This trend is similar to what has been reported for BTV, where the EIP decreases with increasing temperatures [29,30].

While the study by Ruder et al. [26] has provided initial insight into the replication dynamics of EHDV within *Culicoides* midges, fundamental questions of EHDV infection dynamics, including tissue tropism, infection progression, and routes of dissemination remain unresolved. To that end, we conducted an EHDV-2 time course infection study of *C*. *sonorensis* to examine virus infection dynamics by molecular, viral, and immunohistochemical techniques.

## Methods

### *C*. *sonorensis* maintenance and EHDV-2 infections

All experiments were performed with *C*. *sonorensis* females from the Ausman colony [31], which were reared using established protocols [32]. EHDV-2 virus stock (ID no. CC12-304) was prepared from the spleen of an infected white-tailed deer from Kansas in 2012. The virus was isolated in calf pulmonary artery endothelial (CPAE; American Type Culture Collection, Manassas, VA, USA) cells and passed twice in baby hamster kidney (BHK; American Type Culture Collection) cells before purification by centrifugation through a 25% sucrose cushion at 28,000 g for 1 h. For oral infections, adult female midges (3–4 days post eclosion) were allowed to feed for 1 h on a mixture of equal volumes of commercial defibrinated sheep blood (Lampire, Everett, PA, USA) and EHDV-2 virus suspension (10^7.2^ plaque forming units (PFU)/ml in 199E cell culture medium, with a final concentration of 10^6.9^ PFU/ml EHDV-2) using an artificial feeding apparatus with parafilm as a membrane. Engorged females were separated immediately and placed into cages in groups of 80 per cage.

To provide positive controls for immunohistochemical analysis, infections were also performed by intrathoracic (IT) inoculation. For IT inoculation, 3–4 day old female midges were injected as described previously [33] with 50 nl of the same EHDV-2 virus stock. After oral or IT infection, all midges were kept at 25°C and fed 10% sucrose solution ad libitum.

### Sequential insect sampling

Orally infected midges (n = 20 per time point and assay) were collected every 12 h from 0–2 dpf and daily from 3–10 dpf, and processed for immunohistochemistry (IHC), virus isolation and plaque assays, and qPCR. For IHC, females were IT injected with 50 nl EM grade 10% formalin (Thermo Fisher Scientific, Waltham, MA, USA). Subsequently, wings were removed with scissors, and midges were placed overnight in 1.5 ml microcentrifuge tubes containing 500 μl 10% buffered formalin (Thermo Fisher Scientific). Midges were transferred to Tissue-Loc HistoScreen cassettes (two midges per cassette, Thermo Fisher Scientific) and held in 10% buffered formalin for at least 24 h at room temperature until further processing. For virus isolation and plaque assays, female midges were collected and placed individually into 500 μl of midge viral transport medium (199E cell culture medium, 200 U/ml penicillin, 200 μg/ml streptomycin, 100 μg/ml gentamycin, and 5 μg/ml amphotericin B) [34]. Samples were frozen immediately and stored at -80°C until further processing. For qPCR analyses, midges were collected and placed individually in 300 μl Trizol (Ambion, Life Technologies, Carlsbad, CA, USA). Samples were frozen immediately and stored at -80°C until further processing as detailed below.

### Embedding and sectioning

Midges for IHC analyses were embedded in paraffin wax as described previously [35], and stored at room temperature. Embedded midges were serially cut in 5 μm sagittal sections using a Leica RM2235 microtome (Leica, Wetzlar, Germany) with MX35 Premier blades (Thermo Fisher Scientific). Sections were mounted onto positively charged microscope slides (Premiere, C&A Scientific, Manassas, VA, USA) and kept on a slide warmer at 40°C overnight. Midges fed a non-infectious blood meal and IT-infected midges were used as negative and positive controls, respectively, and processed in parallel to the experimental samples.

### IHC

IHC staining was performed by modification of a protocol established previously [35]. Briefly, midge sections were deparaffinized and hydrated with phosphate buffer saline (PBS). Antigens were retrieved by submerging sections in citrate-EDTA buffer (10 mM citric acid, 2 mM EDTA, 0.05% Tween^®^20, at pH 6.2) at 65°C for 30 min. Sections were allowed to cool at room temperature and blocked with 6% casein (Sigma-Aldrich, St. Louis, MO, USA) in PBS for 1 h. Sections were incubated at room temperature for 1 h with a 1:1,000 dilution of a polyclonal rabbit EHDV-2 primary antibody. Sections were then sequentially incubated at room temperature for 1 h with biotinylated rabbit anti-mouse secondary antibody and avidin-biotin alkaline phosphatase, according to manufacturer’s instruction (VECTASTAIN ABC-AP Staining kit, Vector Laboratories, Burlingame, CA, USA). Between each incubation step, samples were washed twice with PBST (PBS, 0.05% Tween^®^20) for 5 min. Sections were incubated with Vector Red chromagen substrate (Vector Laboratories) for 20 min, and counterstained with Meyer’s hematoxylin (Sigma-Aldrich) for 3 min. Sections were covered with CC Mount (Sigma-Aldrich) and air-dried. Coverslips were added using VectaMount (Vector Laboratories).

### Image acquisition and processing

IHC slides were examined for virus-positive (red) staining by light microscopy using a Nikon Eclipse 80i microscope (Nikon, Minato, Tokyo, Japan). Representative images were taken using Leica DFC 7000T camera (Leica), using identical exposure settings across all treatment and control samples. Images were processed in Adobe Photoshop CC 2017 (Adobe Systems, San Jose, CA, USA) using the white balance tool across all treatment and control samples.

### Cytopathic effect and plaque assays

Virus isolation was performed as described previously with minor modification [26]. To isolate infectious virus, midges stored in midge viral transport medium were homogenized individually using a MM40 bead beater (Retsch, Haan, Germany) at 28 beats per second for 2 min with two hollow titanium beads. Samples were centrifuged at 9,615 × g for 4 min and sonicated at 100 A for 5 second bursts, pulsing every 2 seconds for 35 seconds (Q700 Sonicator, Qsonica, Newtown, CT, USA). Sonicated samples were filtered through 0.45 μm DISMIC:13CP filters (Advantec MFS Inc., Toyo Roshi Kaisha Ltd., Bunkyo-ku, Tokyo, Japan). For each sample, 200 μl of undiluted filtered homogenate was added to a monolayer of Vero cells in a 12-well format and incubated at 37°C for 10–14 days in a CO_2_ incubator. Observation of cytopathic effects (CPE) after one passage was used as an indicator of infectious virus within that sample. Infection prevalence was calculated as the proportion of midges whose homogenate showed CPE out of the total number of midges assayed at each corresponding time point.

All homogenates that were virus positive in the CPE assays were analyzed further to determine infectious virus particle (VP) titer by standard plaque assay using Vero cells, with plates incubated at 37°C for 10 days in a CO_2_ incubator. Because of the dilutions used in this assay, the minimum detectable titer was 10^1.39^ PFU/midge.

### RNA extraction and cDNA synthesis

To isolate total RNA from single infected midges sampled over time, midges stored in 300 μl Trizol were homogenized using a motorized pestle and brought to a final volume of 500 μl Trizol. Samples were centrifuged at 9,615 × g for 4 min, and the supernatant was transferred to a 2 ml Heavy Phase Lock Gel tube (5prime, Thermo Fisher Scientific), which was centrifuged for 2 min at 9,615 × g. Next, 60 μl 1-bromo-3chloropropane (BCP) were added to each tube, and samples were centrifuged at 21,100 × g for 15 min. A further 40 μl BCP were added, and samples were centrifuged again at 21,100 × g for 15 min. The aqueous layer was transferred to a fresh 1.5 ml RNAse-free tube and mixed with an equal volume of isopropanol. Samples were incubated for 30 min at room temperature and centrifuged at 21,100 × g for 30 min. The supernatant was removed, and the remaining RNA pellet was washed with 800 μl of 70% ethanol. Pellets were air-dried for 3–4 min, and each pellet was dissolved in 10 μl RNAse-free water. DNA was removed with PerfeCTa DNase I (Quantabio, Beverly, MA, USA) per manufacturer’s instructions, and DNAse I was subsequently heat denatured at 80°C for 15 min. Complementary DNA (cDNA) was synthesized using the qScript XLT cDNA SuperMix (Quantabio) with 200 ng of total RNA as template and a mixture of random hexamers and oligo(dT) primers in a total reaction volume of 20 μl, following the manufacturer’s protocol.

### qPCR

To quantify EHDV-2 load by viral genomic equivalents (GE), qPCR reactions were performed using primers that anneal to the negative sense strand of genomic segment 10 of EHDV-2, which is translated into non-structural protein 3 (Ns3) [36]. Using iQ SYBR Green Supermix (Bio-Rad, Hercules, CA, USA), 5 μl-undiluted cDNA was used as template for each 20 μl reaction. Primers were designed based on published EHDV-2 *Ns3* complete coding DNA sequence (GenBank accession No. KU140932.1) as follows: Ns3_F 5’-CTACCACAGCCGCAATTA-3’, Ns3_R 5’- GCATGTAAACGAGCAAGTATT-3’. To ensure high quality of the extracted total RNA, midge *Elongation Factor 1b* (*EF1b*, GAWM01010754) transcripts were detected using our previously established protocol [28]. All qPCRs were performed with 3 technical replicates per sample and primer set, using previously described PCR parameters [33]. Samples were deemed virus-positive by this method if (1) *EF1b* amplification resulted in a threshold cycle (C_t_) between 21–25, and (2) the viral amplicon from at least two out of three technical replicates produced a smooth, single-peak melt curve, with a fluorescence maximum at 80°C (S1 Table).

### Calculating total VPs by qPCR

Total RNA from the EHDV-2 stock was extracted in 750 μl Trizol as described previously [33] and resuspended in 40 μl RNAse-free water. To precipitate single stranded RNA, an equal volume of 4 M LiCl was added to each sample and incubated for 1 h at -20°C. Samples were centrifuged at 21,100 × g for 1 h at 4°C, and the supernatant was transferred to a new tube. To precipitate viral genomic dsRNA from the supernatant, an equal volume of 8 M LiCl was added to each sample and incubated at -20°C for 1 h. Samples were centrifuged 21,100 × g for 1 h at 4°C, supernatants were removed, and pellets were washed with 800 μl 70% ethanol. Pellets were air-dried for 3–5 min and resuspended in 50 μl RNase-free water. Complementary DNA was synthesized using the qScript XLT cDNA SuperMix (Quantabio) with 200 ng of viral genomic dsRNA as template and a mixture of random hexamers and oligo(dT) primers in a total reaction volume of 20 μl, following the manufacturer’s protocol. The resulting cDNA was serially diluted in RNase-free water [undiluted, 1:2, 1:10, 1:20, 1:50, 1:10^2^, 1:10^3^, 1:10^4^, 1:10^5^].

To obtain a standard curve, the serially diluted cDNA was used as template in qPCR reactions with Ns3 primers as described above. The standard curve was generated by plotting the natural log of the dsRNA amount against the observed C_t_ values for each dilution (S1 Fig). Data were analyzed by linear regression, yielding an equation of y = (-1.253)x + 22.63 (Graphpad Prism 6, GraphPad Software Inc., La Jolla, CA, USA). This equation was used to convert C_t_ values obtained from experimental midge samples (S1 Table) to total viral dsRNA per sample, which was further converted to total number of VPs using the ratio published by Huismans et al. (0.1 fg viral dsRNA: viral genome equivalents of 6 VPs) [37].

### Statistical analyses

All statistical analyses were performed using GraphPad Prism software 6 (GraphPad Software, Inc.). Virus load measured by qPCR, were log10-transformed, assessed for normality using the Shapiro-Wilk normality test (W = 0.6995, *P* < 0.0001), and were analyzed using Kruskal-Wallis across all time points. Virus load measured by plaque assay was converted to two categorical datasets, above and below the limit of detection (10^1.39^ PFU/midge). Chi-squared analysis of these data was performed across three grouped time intervals, which were deemed biologically-relevant in congruence with IHC observations: 0–1.5 dpf (viral ingestion/digestion), 2–5 dpf (midgut-virus interactions), and 6–10 dpf (viral dissemination/transmission). Infection prevalence data were grouped by assay, CPE and qPCR, and analyzed by Chi-squared test across all time points.

## Results

### Tissue tropism of EHDV-2 in *C*. *sonorensis*

**Fig 1:**
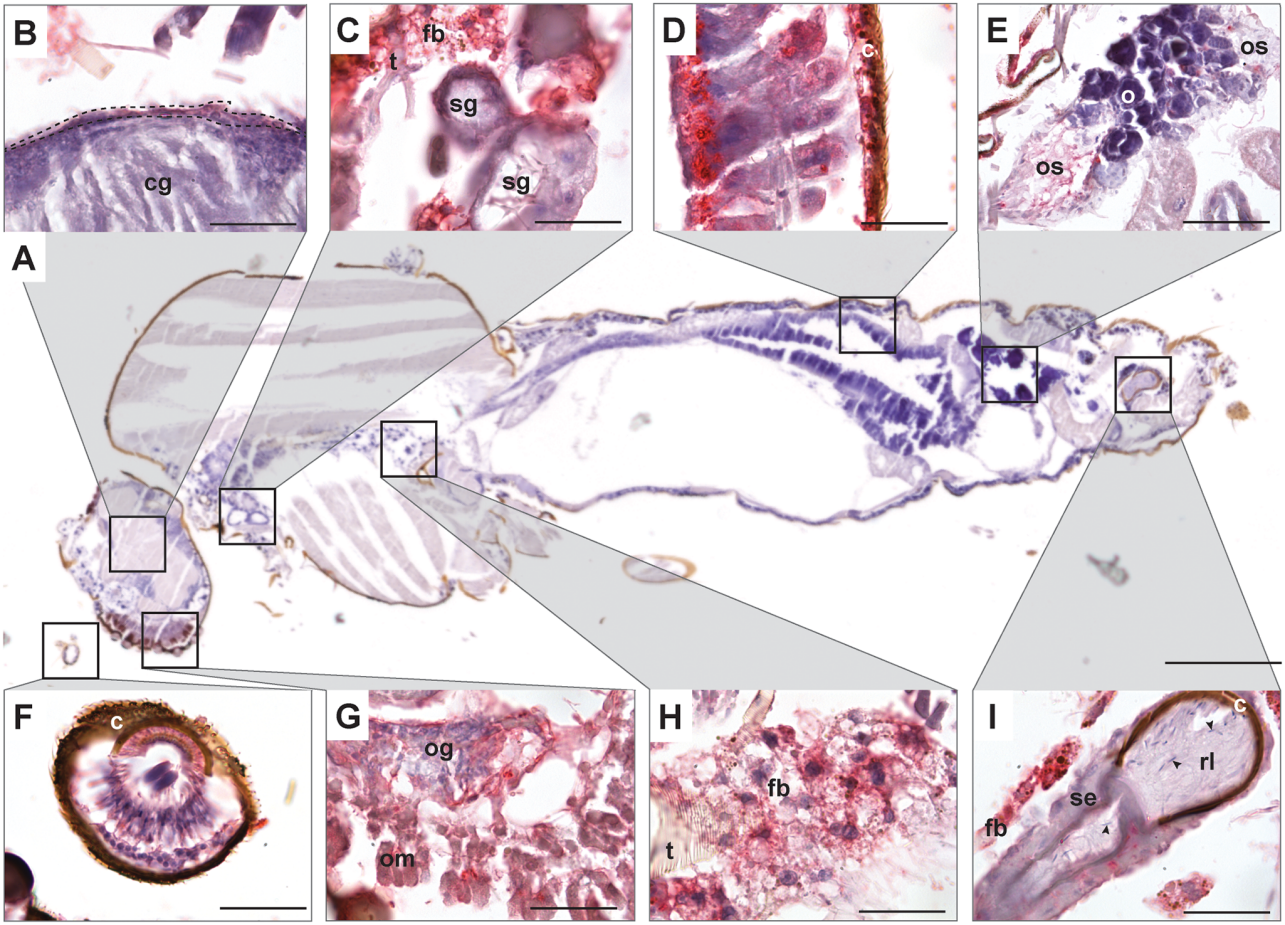
IHC staining of EHDV-2 infected tissues throughout *C*. *sonorensis*. Representative images were taken from orally infected midges at 7–10 dpf. (**A**) The internal anatomy of *C*. *sonorensis* revealed by a hematoxylin-stained sagittal section of the whole midge. No staining for EHDV-2 is provided in this section. Boxes indicate locations of tissues positive for EHDV-2 by IHC staining (red). (**B**) the neural lamella (dotted line) of the cerebral ganglia (cg); (**C**) salivary gland (sg), fat body (fb), trachea (t); (**D**) posterior midgut, cuticle (c); (**E**) ovarian sheath (os), ovariole (o); (**F**) Johnston’s organ (antenna); (**G**) eye containing the ommatidia (om) and optical ganglia (og); (**H**) fat body (fb); and (**I**) spermatheca, which contains the spermathecal epithelia (se), reservoir lumen (rl), and sperm (arrow). All sections were counterstained with hematoxylin (blue) to reveal overall tissue structure. Scale bars: (A) = 200 μm, (B-D and F-I) = 25 μm, and (E) = 50 μm.

To identify the tissues susceptible to EHDV-2 infection within *C*. *sonorensis*, we performed IHC staining on sections of whole-mount infected midges, with representative images taken of infected tissues after dissemination (Fig 1). In disseminated infections, IHC staining of EHDV-2 was observed in the neural lamella of the cerebral ganglia (Fig 1B), salivary glands (Fig 1C), posterior midgut (Fig 1D), Johnston’s organ (antenna; Fig 1F), optic ganglia and ommatidia of the eye (Fig 1G), and fat body (Fig 1H). Interestingly, apparent degradation of the photoreceptor cluster of the ommatidia was associated with EHDV-2 staining in the eye (Fig 1G). No staining was observed in the muscle, anterior midgut, hindgut, and rectum, which suggest these tissues may be refractory to EHDV-2 infection. While tracheal epithelial cells, lumen, and tinidea did not stain for EHDV-2, IHC-positive staining was detected in the tracheoles (S2 Fig). In the ovary, EHDV-2 positive staining was localized to the ovarian sheath but absent in the ovariolar sheath, ovarioles, follicular epithelium, oocytes, and nurse cells (Fig 1E). Epithelial cells of the spermatheca were positive for EHDV-2 staining, but neither the sperm, nor the reservoir lumen, which contains the sperm, were positively stained by IHC (Fig 1I).

### Temporal progression of EHDV-2 infection within *C*. *sonorensis*

**Fig 2:**
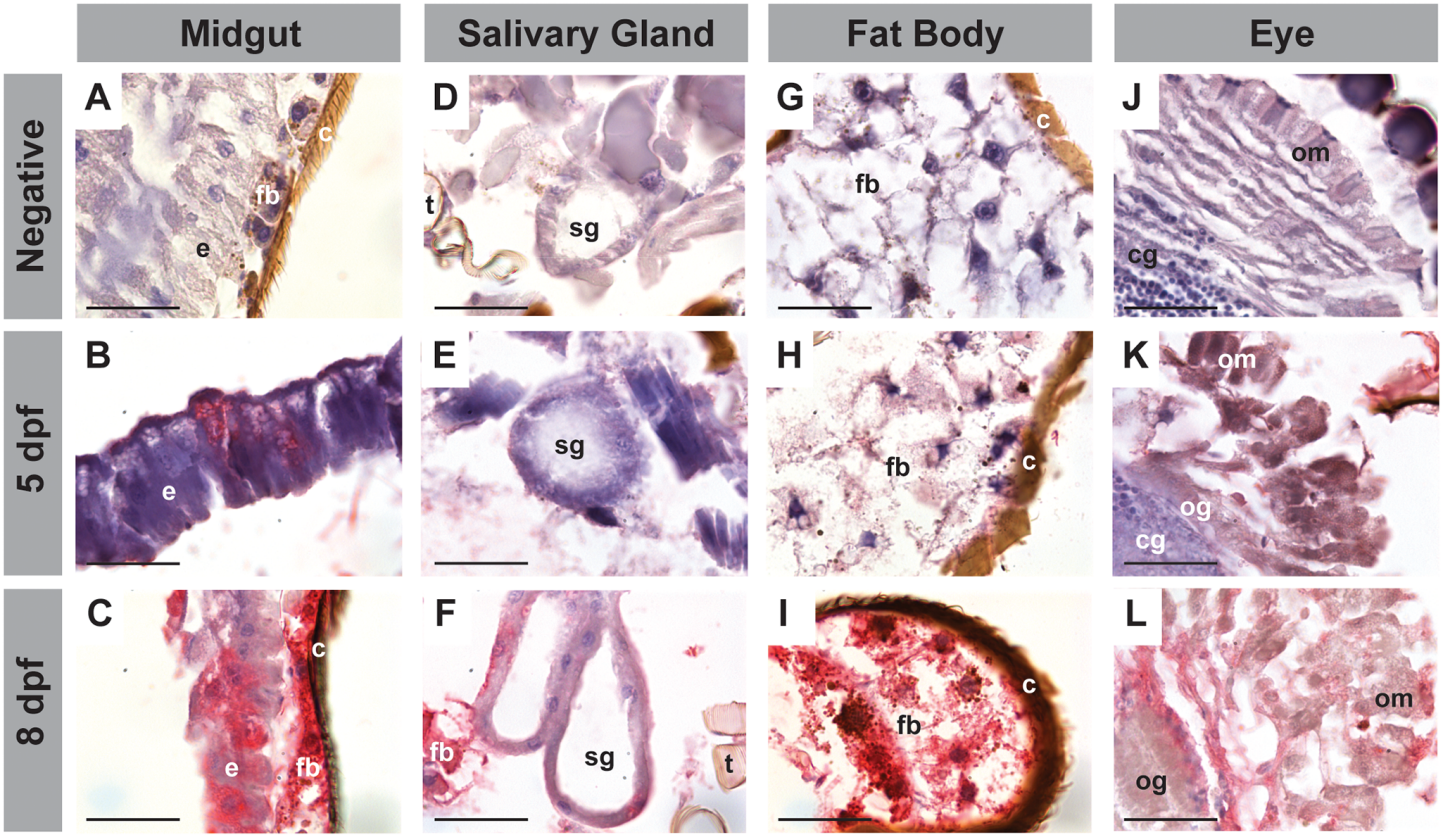
EHDV-2 positive staining pattern in *C*. *sonorensis* by IHC over the course of infection. Increasing intensity of IHC staining (red) showing progression of EHDV-2 infection in tissues over time, with representative images taken at 5 dpf (**B, E, H**, and **K**) and 8 dpf (**C, F, I**, and **L**), compared to negative control tissues (**A, D, G**, and **J**). This increase was observed in all infected tissues: (**A-C**) the midgut [midgut epithelium (e), fat body (fb), cuticle (c)]; (**D-F**) salivary glands (sg), [trachea (t)]; (**G-I**) fat body (fb); and (**J-L**) eye containing the ommatidia (om) and optic ganglia (og) [cerebral ganglia (cg)]. All sections were counterstained with hematoxylin (blue) to reveal overall tissue structure. Scale bars = 25 μm.

To identify the temporal patterns of EHDV-2 infection within the susceptible *C*. *sonorensis* tissues, IHC were performed on midges processed daily 3–10 dpf. We focused on infection progression in the midgut, the primary site of infection following viral ingestion (Fig 2A–2C), and the salivary gland, as these organs are responsible for bite transmission (Fig 2D–2F). In addition, images were taken of tissues that confirmed dissemination: fat body (Fig 2G–2I) and eye (Fig 2J–2L). Following digestion of the blood meal at 2–3 dpf, staining for EHDV-2 was first observed at 5 dpf in foci of the midgut epithelium (Fig 2B). At the same time point, EHDV-2 staining was also observed in salivary glands, fat bodies, and eyes, specifically the ommatidia and optic ganglia (Fig 2E, 2H and 2K). In all midges that were positive for EHDV-2 infection at 5 dpf, staining was more pronounced in the midgut than in other infected tissues (Fig 2B). At time points sampled daily between 6–10 dpf, overall staining intensity was more pronounced in all tissues when compared to staining patterns at 5 dpf (Fig 2C, 2F, 2I and 2L), with the fat body having the strongest and most homogeneous tissue staining (Fig 2I). The midgut and salivary glands both had foci of intense staining, where strongly positive cells were located near non- or weakly-stained cells of the same tissue (Fig 2C and 2F).

### EHDV-2 infection prevalence and titer throughout *C*. *sonorensis* infection

Total and infectious VPs within the sampled midge population were determined throughout the infection time course to reveal trends in EHDV-2 prevalence and virus load (Table 1). At 0 dpf, VPs were detected in 30% and 13% of sampled midges, as determined by CPE and qPCR, respectively. This percentage generally increased through 4 dpf to 53 and 56% of midges, when VPs were isolated and detected by qPCR, respectively. Prevalence levels, measured by either method, appeared to decrease to 20–37% between 5 and 6 dpf and increase again to 50–60% by 10 dpf. At individual time points, up to two-fold differences in prevalence were observed by the two detection methods. While prevalence determined by CPE was not statistically significantly different across the time course (Chi-square = 4.94, d f = 12, *P* = 0.2446), statistically significant changes in the time course of total VP prevalence were detected (Chi-square = 34.46, df = 12; *P* = 0.0006). However, these differences were non-directional, and neither method was consistently more sensitive at detecting EHDV-2.

**Table 1:** Infection prevalence and viral load throughout EHDV-2 infection within the midge.

dpf	Infectious VP		Total VP	
	% CPE positive*	PFU per midge** (n)	% qPCR positive*	Viral Genome Equivalents per midge***
0	30 (6/20)	<1.39 (6)	13 (2/15)	7.81, 8.27
0.5	35 (7/20)	<1.39 (7)	18 (3/17)	6.66, 6.70, 7.10
1	40 (8/20)	<1.39 (7), 2.70	28 (5/18)	7.15, 7.23, 7.27, 7.60, 7.69
1.5	55 (11/20)	<1.39 (10), 2.40	39 (7/18)	6.91, 7.07, 7.32, 7.78, 7.54, 8.03, 8.11
2	39 (7/18)	<1.39 (7)	50 (10/20)	6.56, 7.47, 7.63, 8.11, 8.30, 9.32, 10.10, 11.17, 11.85, 12.61
3	50 (10/20)	<1.39 (10)	41 (7/17)	6.62, 6.74, 7.00, 7.35, 7.72, 8.33, 12.32
4	53 (9/17)	<1.39 (8), 1.88	56 (10/18)	6.72, 7.01, 7.34, 7.42, 7.63, 7.96, 8.28, 11.88, 12.06, 13.85
5	20 (4/20)	<1.39 (1), 2.60, 3.53, 4.01	37 (7/19)	6.38, 6.88, 7.20, 7.29, 7.54, 7.83, 15.41
6	30 (6/20)	<1.39 (6)	31 (5/16)	6.60, 6.73, 7.03, 7.51, 7.64
7	65 (13/20)	<1.39 (13)	56 (9/17)	6.45, 6.87, 6.93, 7.11, 7.18, 7.22, 7.78, 12.36, 15.48
8	35 (7/20)	<1.39 (3), 2.85, 3.68, 5.18, 5.48	45 (8/20)	6.87, 6.94, 7.18, 7.22, 7.78, 12.36, 15.42, 15.48
9	45 (9/20)	<1.39 (7), 4.28, 5.10	90 (18/20)	7.04, 7.11, 7.21, 7.30, 7.38, 7.40, 7.52, 7.62, 7.69, 7.89, 8.05, 8.08, 8.17, 8.26, 8.61, 9.22, 14.34, 14.85
10	50 (10/20)	<1.39 (7), 2.72, 4.90, 5.10	62.5 (5/8)	6.66, 7.53, 8.59, 9.26, 14.49

*no. positive/n

** Log_10_/midge; n is denoted if n > 1. Samples with a PFU of <1.39 were CPE positive, but titer could not be determined as it was below the detection limit.

*** Log_10_/midge. Conversion of C_t_ values to total viral genome equivalents is based on calculation ratios published by Huismans et al. where 0.1 fg viral dsRNA is the genomic equivalent of 6 VPs [32].

**Fig 3:**
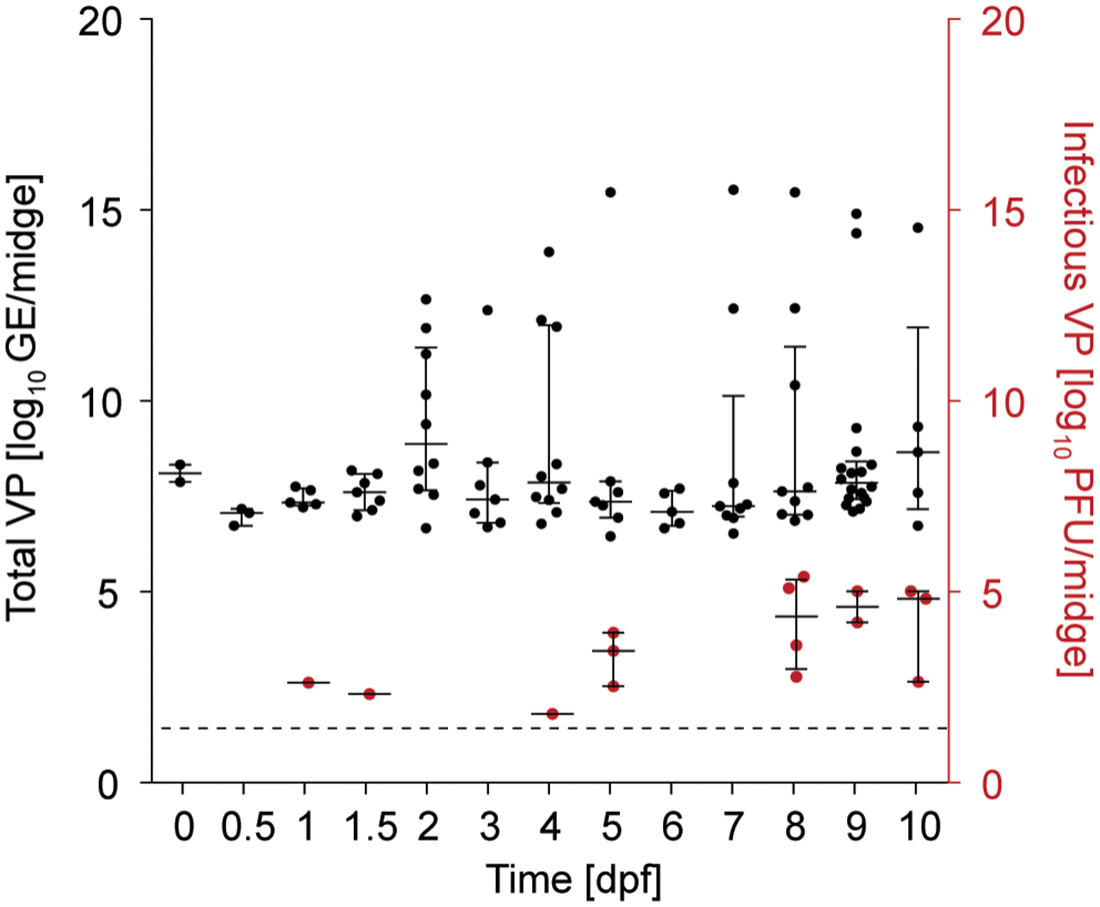
Whole body viral titer over the course of EHDV-2 infection within *C*. *sonorensis*. Graph depicts the viral load per midge over the infection time course. Total VPs (black), were estimated by calculating genome equivalents (GE) by qPCR, and infectious VPs (red with 10^1.39^ PFU/midge limit of detection: dotted line) were calculated by plaque assay. All samples are represented as median and interquartile range. Corresponding raw values are presented in Table 1.

For each EHDV-2-positive midge, the number of total and infectious VPs was determined (Fig 3). Based on the average *Culicoides* blood meal size of 100 nl [38], and titer of the EHDV-2 stock in the infectious blood meal, each midge on average ingested approximately 40 infectious VPs from the EHDV-2 spiked blood meal. Between 0–1.5 dpf, infectious VP load was at or below this average ingestion titer, with VP titers during the first 12 h of infection below the detection limit of 10^1.39^ PFU/midge. Viral load varied across the time course of infection for infectious VP load/midge, and was not statistically significantly different between the time intervals signifying viral ingestion/digestion, midgut-virus interactions, and viral dissemination/transmission (Chi-square = 2.950, df = 2, *P* = 0.2288). Peaks in viral load were observed at 5 and 8 dpf, with similar trends also observed for total viral loads as measured by qPCR. While total viral load was not statistically significantly different across time points (Kruskal-Wallis test H = 15.62, df = 12, *P* = 0.2091), the highest average total VP load was observed at 2, 4, and 10 dpf. Both total and infectious VPs increased steadily from 7 dpf, until reaching their maximum between 8–10 dpf. Throughout the infection time course, total VP load as calculated by qPCR amplification of genomic RNA was consistently greater than infectious VP titer, as calculated by plaque assay with total VP load ranging from 10^7.0^ to 10^8.6^ GE/midge and infectious VP titer ranging from 10^1.88^ to 10^5.48^ PFU/midge. These data suggest that qPCR not only detects released VPs, but also incomplete core particles trapped within infected cells [39].

## Discussion

There are inherent challenges to studying virus-vector interactions such as variation in blood meal virus uptake, variation between individuals within and across biological replicates, as well as detection limits of the assays available. Acknowledging these challenges, we used a multi-pronged approach to analyze the EHDV infection time course in its competent vector *C*. *sonorensis*, using EHDV-2 isolate CC12-304. The time course infection prevalence, dissemination, and viral titer results presented herein provide the first insight into the overall infection dynamics of EHDV in *C*. *sonorensis* midges.

Driven by the small blood meal size, midges take up very few EHDV VPs, at least some of which escape the blood meal during digestion to infect the midgut epithelium. Replication of virus in this first site of infection occurs by 48 hours, the first time point at which we measured an increase in total VP numbers by qPCR. While histological sections showed the blood meal was digested and cleared from *C*. *sonorensis* midguts by three days post ingestion, infectious VP titers remained below the detectable limit for an additional two days, suggesting that virus replication is likely limited and restricted to the midgut epithelial cells. This low viral titer also corresponded with a lack of EHDV staining before 5 dpf. At this time point, infection prevalence reached a temporary plateau that matched endpoint prevalence. EHDV subsequently exited the midgut epithelium and disseminated throughout the body of the midge. Infection of secondary tissues was first detected at 5 dpf, confirming midgut escape and dissemination prior to this time point. Virus replication was observed at all secondary infection sites, reflected in increases in IHC staining and virus titers from 7 dpf onwards. These trends are similar to previous reports that monitored orbivirus load and infection prevalence in *C*. *sonorensis* [40]. Our observations are further supported by published IHC analyses, which detected BTV within the midgut epithelium by 3 dpf [14, 17], and within the salivary glands and saliva between 5–7 dpf [14,18].

Of the *C*. *sonorensis* midges that ingested a virus-spiked blood meal, only 50% were positive for EHDV at 10 dpf, indicating that 50% of midges ingested a viral inoculum titer that was too low to establish infection, cleared the infection by various innate antiviral responses, or were refractory to EHDV [40]. This 50% infection rate was also observed prior to complete blood meal digestion (1.5 dpf) and dissemination (2–4 dpf), suggesting that if virus was ingested, it was cleared in the meal before establishing infection. This observation is distinct from the infection prevalence data observed for BTV-1 [14] and EHDV-7 [26], where initial prevalence at 0 dpf was closer to 100% and a decrease in infection rates was observed between 0 dpf and 3–4 dpf. These data suggest that in contrast to these findings, overall infection rates reported in this current study are likely largely driven by the amount of virus taken up during hematophagy. The effects of midgut infection and escape barriers were possibly reflected in the decreasing trend in viral titers observed at days 3 and 6, respectively.

Orbivirus EIP in *C*. *sonorensis* has been measured previously as the time period between virus uptake and an infectious virus titer larger than 10^2.7^ TCID_50_/midge. This is considered the threshold for vector competence [14,28,41], and is substantiated by detecting VPs in the salivary glands/saliva [14,18]. We observed EHDV in the salivary gland epithelia as early as 5 dpf, the same time point at which infectious VP titers crossed the vector competence threshold for the first time. These data suggest an EHDV-2 EIP in *C*. *sonorensis* at or below 5 days at 25°C, which is slightly shorter than EIPs reported previously for EHDV-1, -2, and -7 (6 dpf) [26]. As the lengths of orbivirus EIP and other arboviruses are temperature dependent [29,30], the short EIP seen in this study could become even more reduced at higher temperatures. Climate change is predicted to precipitate such a phenomenon in *C*. *sonorensis* throughout large regions of their geographic range in the USA [10].

Arboviruses utilize a variety of routes to disseminate from the midgut to susceptible secondary tissues in the insect including the tracheal network, neural network, or hemolymph [42–44]. Based on the observed infection time course of these secondary tissues, EHDV disseminates from the midgut via the hemolymph to infect all susceptible secondary tissues, including the salivary glands, at approximately the same rate. Equivalent EHDV staining was observed across all secondary tissues with no evidence of a specific sequential infection throughout the midge. In contrast, routes of arbovirus dissemination via the tracheal or neural networks result in a characteristic, sequential infection of host tissues over time [42]. For example, vesicular stomatitis virus (VSV) was detected in *C*. *sonorensis* midges, sequentially along the alimentary canal from foregut to midgut to Malpighian tubules before dissemination via hemolymph. Additionally, VSV was shown to disseminate rapidly from midgut epithelial cells to abdominal node neural cell bodies via retrograde axonal transport and progress sequentially down axons to other node cell bodies via anterograde transport [35]. However, EHDV staining was not associated with the neuronal ganglia, and infection was limited to the neural lamella, which serve as the insect blood-brain barrier by lining neuronal tissues [45]. Furthermore, tracheal route for EHDV dissemination is unlikely, as EHDV-2 staining was not detected in tracheal epithelia or lumen and limited only to tracheoles. Together, these observations strongly suggest that EHDV, similar to BTV [14], relies on the hemolymph to reach susceptible secondary tissues.

Tissue tropism can be used to infer physiological consequences of virus infection. EHDV staining was observed in the midge ommatidia, optic ganglia, and Johnston’s organ, which provide visual and auditory perception [46,47]. EHDV infection and replication was associated with damage to the ommatidia, which could result in impaired function and subsequent behavioral changes of *C*. *sonorensis*. Previous studies observed BTV-1 and -17 infected *C*. *sonorensis* ommatidia [14,48] and light aversion behavior was attributed to infection of these vision organs [48]. Some impact of virus infection on vector behavior, such as host-seeking, has also been suggested for mosquito-borne viruses including dengue [49–51]. Future studies are required to examine EHDV infection-related damage of sensory tissues and determine the potential consequences on altered sensory perception in *C*. *sonorensis* host-seeking behavior.

Analysis of viral tissue tropism also revealed EHDV infection of *C*. *sonorensis* female reproductive organs. EHDV staining was associated with the epithelia of the *C*. *sonorensis* spermatheca, which is structurally similar to other dipteran vectors [52–54]. In the ovaries, EHDV staining was limited to the ovarian sheath, which does not come into direct contact with the developing oocyte [55], and was absent from the ovariolar sheath, ovarioles, follicular epithelium, oocytes, and nurse cells. These observations suggest a barrier to EHDV infection of *C*. *sonorensis* ovaries. Previous studies detected BTV in the ovarian sheath, within the immature yolk bodies and vitelline membrane of the developing *Culicoides* oocyte, as well as on eggs oviposited by BTV-infected females [58–61]. Nevertheless, these studies found no evidence for vertical transmission of BTV by *Culicoides* [56–59]. Considering our findings along with these published data, it is unlikely that vertical transmission of EHDV occurs in its vector, *C*. *sonorensis*. Given the role of the spermatheca in sperm survival and reproductive success [60], future studies should investigate the potential effects of EHDV spermathecal epithelium infection on reproduction.

In summary, this study was the first to examine EHDV infection dissemination, infection prevalence, and viral titer simultaneously within *C*. *sonorensis* over the course of infection. The viral titer and infection prevalence data reported here showed similar trends in EHDV infection observed by Ruder et al. [26] using the same serotype, and linked these trends with the temporal-spatial fate of the virus. Similar to BTV, EHDV disseminates through the hemolymph of *C*. *sonorensis* to the salivary glands by 5 dpf, suggesting an EIP of 5 d or shorter. In addition, this study is the first to identify the physiological basis for the lack of EHDV vertical transmission in its insect vector. Overall trends in VP load and infection prevalence were similar between qPCR and plaque assays, confirming that the molecular assay, described herein, can be used to monitor EHDV-2 infection progression in its vector over time. Together, these findings fill important gaps in our knowledge of EHDV interactions with its vector.

## Supporting information

S1 Fig Viral dsRNA qPCR standard curve of EHDV-2 genomic Ns3. Cycle thresholds (C_t_) are plotted against the natural log of viral dsRNA concentration. The equation of the linear regression (solid line) was used to determine the amount of viral genomic dsRNA per midge and converted to viral genome equivalents as a measure for the number of total viral particles per midge according to Huismans et al [32].
(TIF)

S2 Fig IHC staining of EHDV-2 associated with tracheoles in *C*. *sonorensis*. IHC staining (red) indicating EHDV-2 infection is associated with the tracheoles (black line), while not found in the tracheal tinidea (t), lumen, or epithelia (dotted line). Scale bar = 25 μm.
(TIF)

S1 Table C_t_ values and genomic equivalence data from samples positive for EHDV-2 by qPCR. (XLSX)
