# Supplementary figures and images for: Dynamics of epizootic hemorrhagic disease virus infection within the vector, Culicoides sonorensis (Diptera: Ceratopogonidae)
Source: PLoS One. 2017 Nov 27;12(11):e0188865. doi: 10.1371/journal.pone.0188865 (PMC5703522; doi:10.1371/journal.pone.0188865)

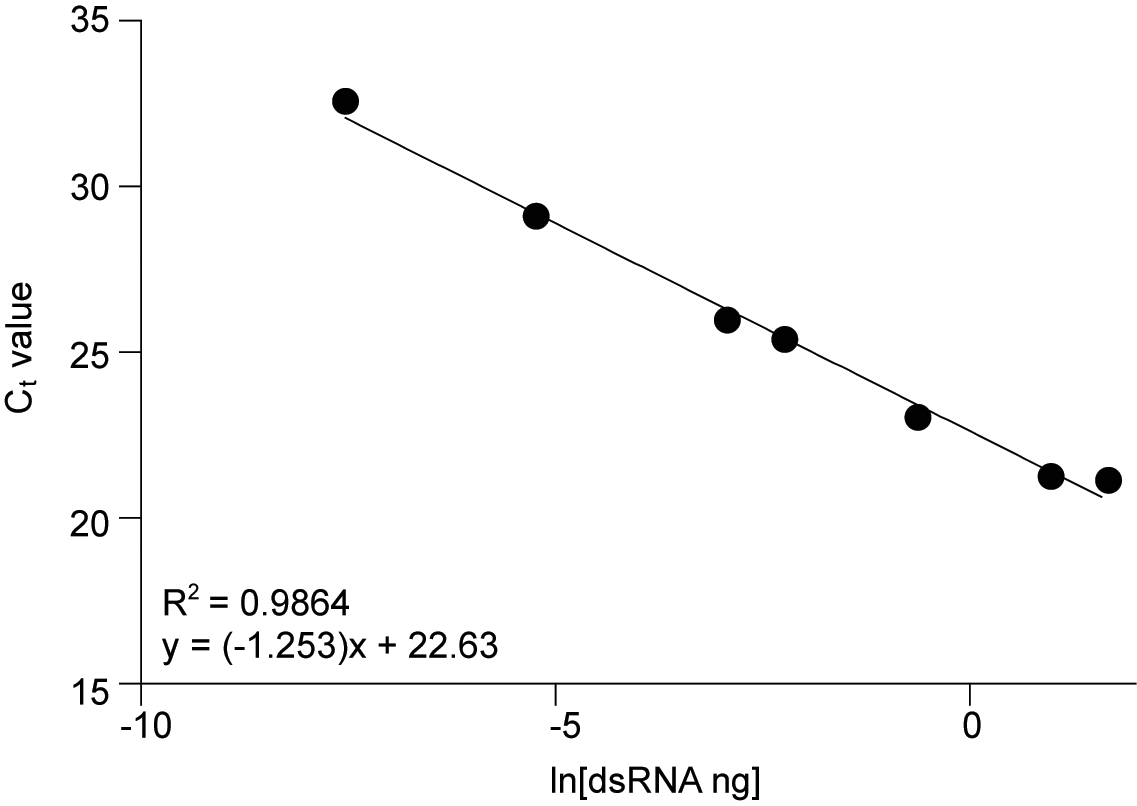

Supplement: S1 Fig — Cycle thresholds (Ct) are plotted against the natural log of viral dsRNA concentration. The equation of the linear regression (solid line) was used to determine the amount of viral genomic dsRNA per midge and converted to viral genome equivalents as a measure for the number of total viral particles per midge according to Huismans et al [32]. (TIF) [file pone.0188865.s001.tif]

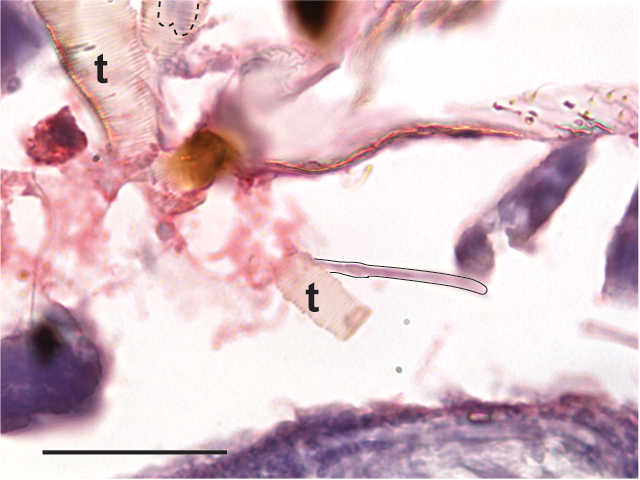

Supplement: S2 Fig — IHC staining (red) indicating EHDV-2 infection is associated with the tracheoles (black line), while not found in the tracheal tinidea (t), lumen, or epithelia (dotted line). Scale bar = 25 μm. (TIF) [file pone.0188865.s002.tif]
